# Probing and regulating the activity of cellular enzymes by using DNA tetrahedron nanostructures†
†Electronic supplementary information (ESI) available: Experimental section and supplementary tables and figures. See DOI: 10.1039/c9sc01912j


**DOI:** 10.1039/c9sc01912j

**Published:** 2019-05-06

**Authors:** Yi Zhang, Yingnan Deng, Congshan Wang, Lidan Li, Lida Xu, Yingjie Yu, Xin Su

**Affiliations:** a College of Life Science and Technology , Beijing University of Chemical Technology , Beijing 100029 , China . Email: xinsu@mail.buct.edu.cn; b Department of Biomedical Engineering , Tufts University , Medford , MA 02155 , USA . Email: yuyingjie312@outlook.com

## Abstract

Given the essential role of apurinic/apyrimidinic endonuclease (APE1) in gene repair and cancer progression, we report a novel approach for probing and regulating cellular APE1 activity by using DNA tetrahedrons. The tetrahedron with an AP site-containing antenna exhibits high sensitivity and specificity to APE1. It is suitable for APE1 *in vitro* detection (detection limit 5 pM) and cellular fluorescence imaging without any auxiliary transfection reagents, which discriminates the APE1 expression level of cancer cells and normal cells. In contrast, the tetrahedron with an AP site on its scaffold exhibits high binding affinity to APE1 but limits enzymatic catalysis making this nanostructure an APE1 inhibitor with an IC_50_ of 14.8 nM. It suppresses the APE1 activity in living cells and sensitizes cancer cells to anticancer drugs. We also demonstrate that the APE1 probe and inhibitor can be switched allosterically *via* stand displacement, which holds potential for reversible inhibition of APE1. Our approach provides a new way for fabricating enzyme probes and regulators and new insights into enzyme–substrate interactions, and it can be expanded to regulate other nucleic acid related enzymes.

## Abstract 2

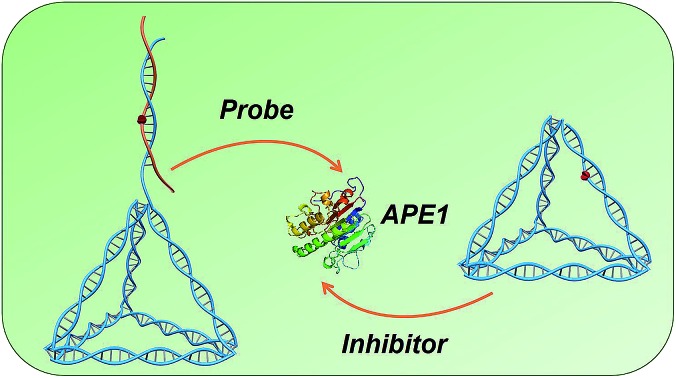
Given the essential role of apurinic/apyrimidinic endonuclease (APE1) in gene repair and cancer progression, we report a novel approach for probing and regulating cellular APE1 activity by using DNA tetrahedrons.

## Introduction

Enzymes play a vital role in cellular activities. Apurinic/apyrimidinic endonuclease 1 (APE1) is a multifunctional enzyme involved in the base excision repair (BER) pathway, which accurately removes damaged bases and guarantees genomic integrity.^1^ APE1 is a prerequisite for the repair of DNA damage in both the short-patch and long-patch pathways of BER, although each pathway employs different enzymes to complete repair subsequent to APE1-mediated cleavage.^2^ APE1 is responsible for >95% of apurinic/apyrimidinic (AP) site processing in mammalian cells.^3^ APE1 is also involved in the regulation of transcription as well as RNA transcription/modulation. Dysregulation of APE1 has been demonstrated to be associated with a couple of diseases such as cancer,^4^ neurodegenerative diseases^5^ and cardiovascular disorders.^6^ Abnormal expression and subcellular distribution of APE1 have been linked to tumor metastasis.^7^ From a therapeutic perspective, APE1 has drawn significant attention as an emerging target for some cancer types.^4^ This motivates fabricating molecular tools to probe and regulate the cellular APE1 activity. DNA based molecular probes have been developed for measuring APE1 activity,^8^–^10^ but rarely used for intracellular enzyme regulation. Some small molecules as conventional APE1 inhibitors are proven effective in some cancer cell types.^11^–^13^ However, they always suffer from poor sensitivity and specificity as well as multiple drug resistance (MDR).^14^

Watson–Crick base pairing enables the ‘bottom-up’ construction of DNA structures with high controllability and precision at the nanoscale in a programmable manner.^15^,^16^ Owing to the addressability and programmability, DNA nanostructures have been utilized to organize a variety of functional components.^17^,^18^ DNA tetrahedrons are versatile 3D frameworks consisting of four single-stranded DNAs (ssDNA).^19^,^20^ Due to their well-defined size and excellent biocompatibility, DNA tetrahedrons have been widely utilized in biosensors, nanodevices, and drug delivery.^21^ For instance, Fan and colleagues constructed a series of DNA tetrahedron-based assays for a couple of biomarkers.^22^,^23^ It is generally accepted that DNA tetrahedrons have a promising ability of cellular uptake without any auxiliary materials.^24^,^25^

Here, inspired by the properties of DNA nanostructures, we demonstrate a DNA tetrahedron-based approach for probing and regulating the APE1 activity in living cells. Distinct from conventional molecular probes and inhibitors, the designed DNA tetrahedrons act as a probe and inhibitor which can be switched by translocating the AP site. The AP site on the tetrahedron antenna (OUT-tetrahedron) can be rapidly cleaved by APE1. The OUT-tetrahedron exhibits high sensitivity and specificity towards APE1, reaching a detection limit as low as 5 pM. It is used for *in situ* fluorescence imaging of APE1 in living cells without any auxiliary transfection reagent. In contrast, the tetrahedron containing AP site on its scaffold (IN-tetrahedron) allows for efficient APE1 binding but inhibits catalysis making this nanostructure a putative APE1 inhibitor. The IN-tetrahedron has a lower IC_50_ of 14.8 nM for APE1 inhibition than many small-molecule inhibitors. It suppresses APE1 activity in living cells. Taking advantage of the inhibitory effect of the IN-tetrahedron, we demonstrate the potentiation of cytotoxicity of anticancer drugs by the IN-tetrahedron, and the dosage of the IN-tetrahedron is lower than previously reported small-molecule inhibitors. This work provides a novel approach to regulate enzyme activity and new insights into enzyme–substrate interactions, and it can find broad applications in gene repair regulation, enzyme inhibition, and cancer therapy.

## Results and discussion

### Design and principle of the AP site-containing DNA tetrahedrons

**Fig. 1:**
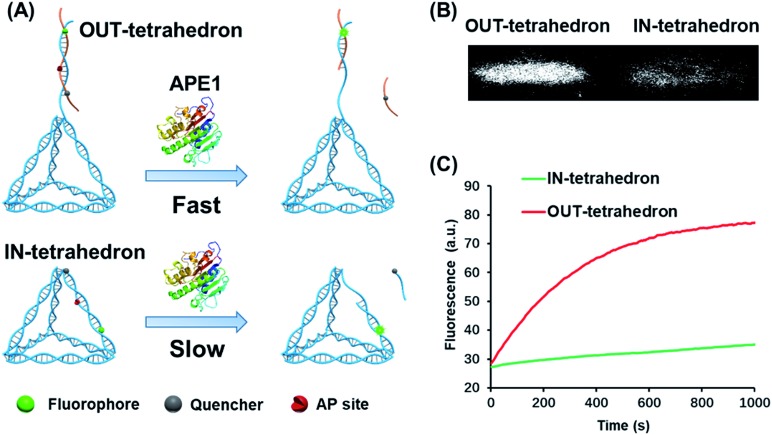
Reactivity of APE1 towards the two designed DNA tetrahedrons. (A) Schematic illustration of the APE1 enzymatic reaction of the DNA nanostructures, OUT-tetrahedron and IN-tetrahedron. The cleavage of the AP site on the scaffold and antenna yields different rates. (B) The native PAGE gel analysis of the reaction products of the two DNA nanostructures which are labeled with a fluorophore and quencher as in panel A. The gel was not stained, and the bands represent the nanostructures. (C) Real-time monitoring of the reaction of the two DNA nanostructures. The concentration of DNA tetrahedrons and APE1 is 100 nM and 0.16 nM, respectively. The enzymatic reactions were performed in TAE-Mg^2+^ buffer (ESI†) in which APE1 exhibits similar activity to that in the recommended buffer (50 mM potassium acetate, 20 mM tris-acetate, 10 mM magnesium acetate, 1 mM DTT, pH 7.9) (Fig. S3, ESI†).

The structure of the OUT-tetrahedron and IN-tetrahedron is shown in Fig. 1A. To prevent the autonomous cleavage of the AP site at high temperature, we annealed the single strands at 80 °C to prepare the DNA tetrahedrons (for sequences see Table S1†). The as-prepared nanostructures were purified by ultrafiltration. The assembly of DNA tetrahedrons was first confirmed by native gel electrophoresis (Fig. S1, ESI†), and the clear single bands suggest the high purity and stability of the DNA nanostructures as previously reported.^26^ An atomic force microscope (AFM) was also used to further confirm the formation and size of the nanostructures (Fig. S2, ESI†). The comparison of the tetrahedrons with/without the AP site suggests that the presence of the AP site on the scaffold does not affect the stability of the nanostructures (Fig. S1, ESI†). To examine the enzyme activity, we labeled the tetrahedrons with a fluorophore and quencher. The APE1 cleaves the AP site yielding a single strand nick. A quencher labeled 8-mer single stranded fragment is released from the two DNA tetrahedrons because the 8-mer product cannot remain base-paired to its complement at 37 °C. The tetrahedrons are therefore fluorescent. The band of the reaction product of the OUT-tetrahedron is much brighter than that of the IN-tetrahedron implying higher enzyme activity on the OUT-tetrahedron (Fig. 1B). By monitoring the reaction in real time, we found that the fluorescence of the OUT-tetrahedron increases more rapidly than that of the IN-tetrahedron (Fig. 1C). Accordingly, the location of the AP site on the tetrahedrons has a significant influence on the endonuclease activity of APE1.

**Fig. 2:**
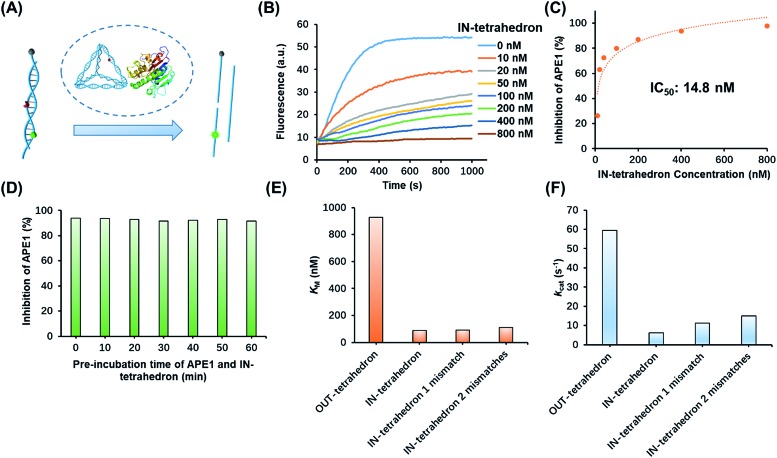
Inhibition of APE1 activity by the IN-tetrahedron. (A) The measurement of APE1 activity by using a dual-labeled probe (for the sequence see Table S1†). (B) Fluorescence signal of the digestion rates of the dsDNA probe by the IN-tetrahedron bound APE1. The IN-tetrahedron concentration is varied, and the APE1 concentration is fixed at 0.16 nM. (C) Quantification of the data set from panel B showing the percentage inhibition of APE1 as a function of IN-tetrahedron concentration. (D) The inhibition effect is not affected by the pre-incubation time of APE1 and the IN-tetrahedron (for raw fluorescence signals see Fig. S4, ESI†). The concentration of APE1 and the IN-tetrahedron is 0.16 nM and 400 nM, respectively. *K*_m_ (E) and *k*_cat_ (F) of different DNA tetrahedrons. All mismatches are adjacent to the AP site. For raw fluorescence signal and Lineweaver–Burk plots see Fig. S5, ESI.†

The suppression of nuclease activity by DNA nanostructures was previously reported.^27^,^28^ The resistance to enzymatic degradation was believed as a result of a combined inhibition of both binding and catalytic activity.^27^ To test whether the enzyme binding is prohibited, we first incubated the IN-tetrahedron with APE1, and used a dual-labeled double-stranded DNA (dsDNA) as a probe to measure the activity of APE1 (Fig. 2A) which can be rapidly digested by APE1 (Fig. 2B). If the binding affinity of APE1 and the IN-tetrahedron is low, the dsDNA probe will also be digested rapidly. Interestingly, slow digestion of the probe was observed when APE1 was pre-incubated with the IN-tetrahedron. The inhibition of APE1 activity is dependent on the IN-tetrahedron concentration (Fig. 2B). The IN-tetrahedron has an IC_50_ of 14.8 nM for the inhibition of APE1 (Fig. 2C) which is much lower than that of previously reported small molecule inhibitors.^11^,^29^,^30^ Moreover, the inhibition is independent on the time of pre-assembly of the IN-tetrahedron and APE1 (Fig. 2D). This implies that the binding of APE1 and the IN-tetrahedron is fast and tight.

### Mechanistic insights into the APE1 enzyme activity on different DNA tetrahedrons

To further investigate the distinct APE1 activity on different DNA tetrahedrons, we measured the kinetic parameters of enzymatic cleavage by ensemble fluorescence assay. The Michaelis constant *K*_M_ and the catalytic rate constants *k*_cat_ are derived from Lineweaver–Burk plots (Fig. S5, ESI†). As shown in Fig. 2E and F, the *k*_cat_ of the OUT-tetrahedron is 9.4 times higher than that of the IN-tetrahedron, and its *K*_M_ is 10.5 times higher than IN-tetrahedron's *K*_M_. Small *K*_M_ and *k*_cat_ indicate high binding affinity and limited catalytic capability, which makes the IN-tetrahedron a putative APE1 inhibitor. A previously reported crystal structure reveals that the specific binding of APE1 to extrahelical AP sites derives from a hydrophobic pocket bordered by three amino residues, which pack with the hydrophobic side of the AP deoxyribose.^31^ The rigid scaffold of DNA tetrahedrons promotes the AP site flipping which is beneficial for APE1 binding. The bending of the substrate DNA is required for effective catalysis. The rigid structure of the IN-tetrahedron restricts the bending resulting in limited catalysis. We introduced mismatches which are adjacent to the AP site of the IN-tetrahedron to make a relatively flexible recognition site for APE1. *k*_cat_ increases as the number of mismatches increases, but there are no significant changes in *K*_m_ (Fig. 2E and F). This result suggests that the presence of mismatches that are adjacent to the AP site in the IN-tetrahedron also permits high enzyme binding affinity and improves the enzymatic catalysis. Based on these results, we speculated that the OUT-tetrahedron and IN-tetrahedron can serve as an APE1 probe and inhibitor, respectively.

### Transition of the APE1 probe and inhibitor by toehold strand displacement

**Fig. 3:**
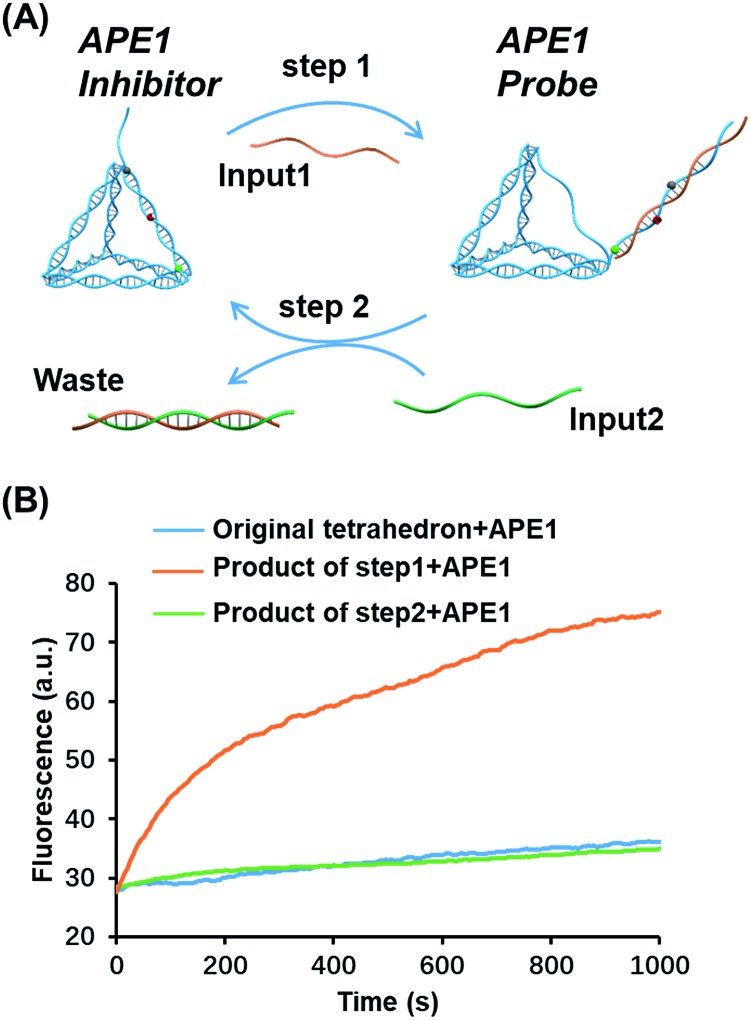
Translocation of the AP site by toehold strand displacement. (A) Step 1: input 1 as an invader strand to initiate strand displacement to switch the AP site from the scaffold to the antenna. Step2: input 2 as an invader strand to neutralize input 1 to re-locate the AP site. The duplex of the two inputs is the waste. (B) Fluorescence signals of different structures reacting with APE1, the original tetrahedron (blue), the structure after the first-step strand displacement (orange), and the structure after the second-step strand displacement (green). The enzyme activity was measured in 40 min allowing the completion of strand displacement. The concentration of the inputs and tetrahedron is 100 nM, and that of APE1 is 0.16 nM.

Toehold strand displacement as a central reaction of DNA nanotechnology has been widely used for the re-configuration of DNA nanostructures.^32^,^33^ It is facile to design this reaction to translocate the AP site on the tetrahedron since the scaffold of the DNA tetrahedron is double-stranded. As illustrated in Fig. 3A, input 1 as an invader strand binds with the strand with an overhang to replace its complement. The AP site is switched from the scaffold to the antenna. Similarly, input 2 binds with input 1 to release the tetrahedron, and then the AP site returns to its original position. The duplex of input 1 and input 2 is the waste of step 1 and 2. According to the kinetics of toehold strand displacement, 40 min is enough for 80% completion of the reaction.^32^ We therefore measured the enzyme reactivity 40 min after introducing the inputs. Consistent with the trend in Fig. 1C, the enzymatic activity on the original tetrahedron and the product of step 2 is low, whereas the product of step 1 is rapidly digested by the enzyme (Fig. 3B). In the overall operation, the activity of APE1 is allosterically regulated by translocating its recognition site *via* strand displacement. Input 1 and input 2 serve as an activator and de-activator for APE1. We demonstrate that it is possible to rationally introduce Nature's solutions, such as allostery, into functional DNA nanostructures regulating the gene repair activity. Moreover, toehold strand displacement provides a solution for reversible inhibition of APE1.

### Highly sensitive and specific APE1 detection by the OUT-tetrahedron

**Fig. 4:**
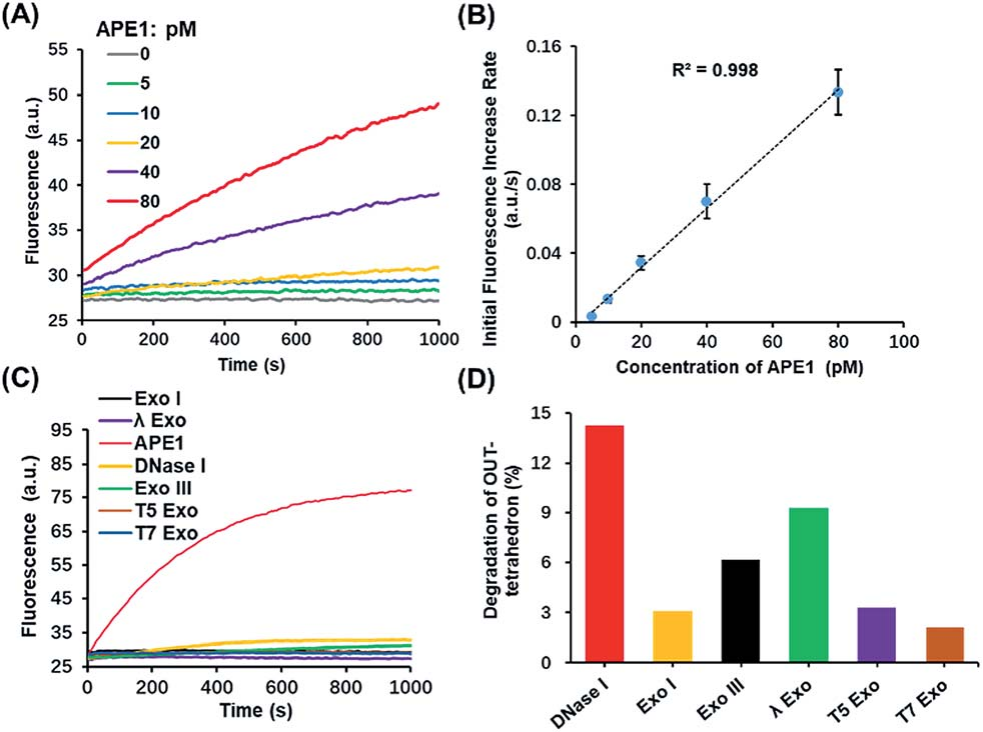
Highly sensitive and selective *in vitro* detection of APE1 by using the OUT-tetrahedron. (A) Fluorescence signal using the OUT-tetrahedron and different concentrations of APE1. (B) Calibration curve of APE1 assay. Linear fit yields *R*^2^ values > 0.99. (C) Fluorescence signals of the OUT-tetrahedron digested by APE1 and non-specific enzymes. (D) Percentage degradation of the OUT-tetrahedron by the non-specific enzymes for 3 h.

Based on the above results, the OUT-tetrahedron is a promising probe to quantify the APE1 activity. The fluorescence signal of the IN-tetrahedron increases as a function of APE1 concentration (Fig. 4A). The initial rates of the fluorescence–time curves show a good linear relationship with the APE1 concentration in the range from 5 to 80 pM (Fig. 4B). The detection limit was determined to be as low as 5 pM. DNA molecular probes are vulnerable in real biological samples because of the nonspecific degradation of enzymes such as DNase.^34^,^35^ Phosphorothioate (PS) modification is always utilized to suppress the nuclease activity.^36^ This strategy is suitable for the protection of the probes from nucleic acids, but not suitable for the probe of enzyme activity because the target enzyme can be also affected. To test the specificity of the OUT-tetrahedron towards APE1, we examined the possible nonspecific interactions of the OUT-tetrahedron with some nucleases that can digest dsDNA or ssDNA. No significant fluorescence increase was observed when the OUT-tetrahedron was incubated with these nucleases (Fig. 4C). The degradation of the antenna of the OUT-tetrahedron by these enzymes is below 15% after 3 h (Fig. 4D). This confirms the high specificity of the OUT-tetrahedron to APE1. The non-specific nucleases can be categorized into two classes, exonucleases and endonucleases. In our system, the resistance of exonuclease activity is mainly attributed to the designed overhangs of the antenna domain because the exonucleases always recognize the blunt and recessive end.^37^–^39^ On the other hand, it is always difficult to protect the DNA probe from non-specific endonucleases such as DNase I because the entire DNA backbone can be attacked by these nucleases. As previously reported, there is a substantial delay before any degradation of the external part of a DNA tetrahedron.^40^ Unlike the mechanism of exonucleases, the tetrahedron scaffold protects its antenna from endonuclease degradation to some extent. Accordingly, the rational design of the tetrahedron probe allows for high sensitivity and specificity to APE1 with potential to probe the intracellular AP endonuclease activity.

### Detecting and regulating endogenous APE1 in living cells

**Fig. 5:**
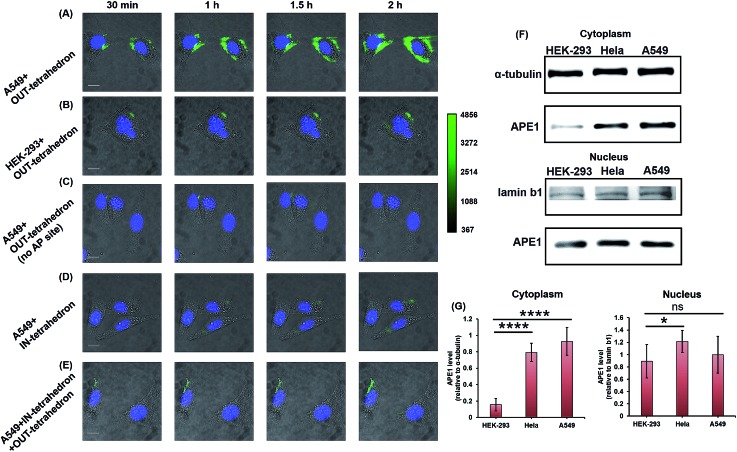
Probing and regulating the APE1 activity in living cells. HILO fluorescence imaging of the cellular APE1 activity of A549 (A) and HEK-293 (B) cells by using the OUT-tetrahedron. (C) Fluorescence imaging of the A549 cell with the OUT-tetrahedron (no AP site) at different times. (D) IN-tetrahedron does not exhibit significant fluorescence in the cytoplasm of the A549 cell consistent with the *in vitro* assay. (E) Probing the APE1 activity of the IN-tetrahedron-pretreated A549 cell by using the OUT-tetrahedron. 400 nM IN-tetrahedron was pre-incubated with the A549 cells for 4 h. For all fluorescence imaging experiments, the nucleus was stained with Hoescht33342, and the OUT-tetrahedron (labeled with Cy3 and BHQ2) concentration is 100 nM. Scale bar: 5 μm. Note that we used cell culture media to carry the DNA tetrahedrons which are in TAE-Mg^2+^ buffer. To evaluate the potential effect of the TAE-Mg^2+^ buffer, we compared the tetrahedrons in 1× PBS and in the TAE-Mg^2+^ buffer by the assay of panel A. No significant difference was found (Fig. S8, ESI†). (F) Western blotting analysis of cytoplasmic and nuclear APE1. (G) Relative quantification of the western blotting of APE1. ns *p* > 0.05, *p < 0.05, **p < 0.01, ***p < 0.001, and ****p < 0.0001.

APE1 has long been believed to be located in the nucleus, and cytoplasmic expressions were found in several types of cancer including epithelial ovarian cancer,^41^ hepatocellular carcinoma,^42^ and non-small-cell lung cancer.^43^ The cytoplasmic localization was associated with the poor prognostic factors of cancers.^44^,^45^ An immunohistochemical stain was always utilized for evaluating the expression level and localization of APE1 in cells.^46^,^47^ But it is labor intensive, slow, and difficult to automate. The capability of cellular uptake of some DNA nanostructures permits intracellular applications.^21^,^48^ DNA tetrahedrons can be rapidly internalized in living cells through a caveolin-dependent pathway.^25^ To probe the APE1 activity in living cells, we incubated 100 nM OUT-tetrahedron with a human lung cancer cell A549 and human embryonic kidney cell HEK-293T. The cytoplasm of the A549 cell exhibits bright fluorescence within 2 h (Fig. 5A). In contrast, the fluorescence of the HEK-293 cell is relatively weak (Fig. 5B). This suggests a higher expression level of APE1 in cancer cells. To confirm the specificity, we introduced the OUT-tetrahedron without the AP site into A549 cells. No significant fluorescence was found when using the AP-site free probe (Fig. 5C). Therefore, the strong fluorescence can be attributed to APE1 mediated fluorescence enhancement. To demonstrate the general feasibility of the tetrahedron probe, the APE1 level in a HeLa cell was also detected, showing a high expression level as the A549 cell (Fig. S6, ESI†). The tetrahedron probes mainly reflect the cytoplasmic APE1 since the tetrahedron cannot enter into the nucleus. *tert*-Butyl-hydroperoxide (TBHP) as a reactive oxygen species (ROS) generator is used to increase DNA damage inducing the overexpression of APE1.^49^ The small molecule 7-nitroindole-2-carboxylicacid (NCA) shows a strong inhibition effect of the cellular APE1 activities.^29^,^50^ Compared with the untreated A549 cells, stronger and weaker fluorescence was observed in the TBHP and NCA treated cells, respectively (Fig. S7, ESI†). Interestingly, we found green fluorescence in both the nucleus and cytoplasm in the presence of TBHP (Fig. S7A, ESI†). TBHP is able to induce apoptosis and necroptosis^51^ potentially increasing the permeability of the nuclear membrane allowing for the detection of nuclear APE1. To further confirm the probe specificity and the APE1 distribution, we carried out western blotting. As shown in Fig. 5F, APE1 was found in the cytoplasm of all cell lines, and it is highly overexpressed in cancer cells, which is consistent with the fluorescence imaging. The difference of cytoplasmic APE1 between cancer cells and normal cells is more significant than that of nuclear APE1 (Fig. 5G). Overall, the OUT-tetrahedron is able to precisely reflect the level of intracellular APE1 activity, particularly to distinguish the differential cytoplasmic level of APE1 in cancer and normal cells.

As mentioned above, the IN-tetrahedron can bind with APE1 and inhibit its catalytic activity. As expected, dim fluorescence was observed when the A549 cells were incubated with the fluorophore and quencher labeled IN-tetrahedron (Fig. 5D). In order to test whether the IN-tetrahedron can be used as an APE1 inhibitor in living cells, A549 cells were incubated with 400 nM IN-tetrahedron for 2 h followed by incubation with 100 nM OUT-tetrahedron that serves as an APE1 probe. Consistent with the assay using NCA as the inhibitor, dim fluorescence was also observed in the IN-tetrahedron pretreated cells (Fig. 5E). This confirms that the OUT-tetrahedron can serve as an APE1 inhibitor in living cells.

### IN-tetrahedron sensitizing cancer cells to cytotoxic agents

**Fig. 6:**
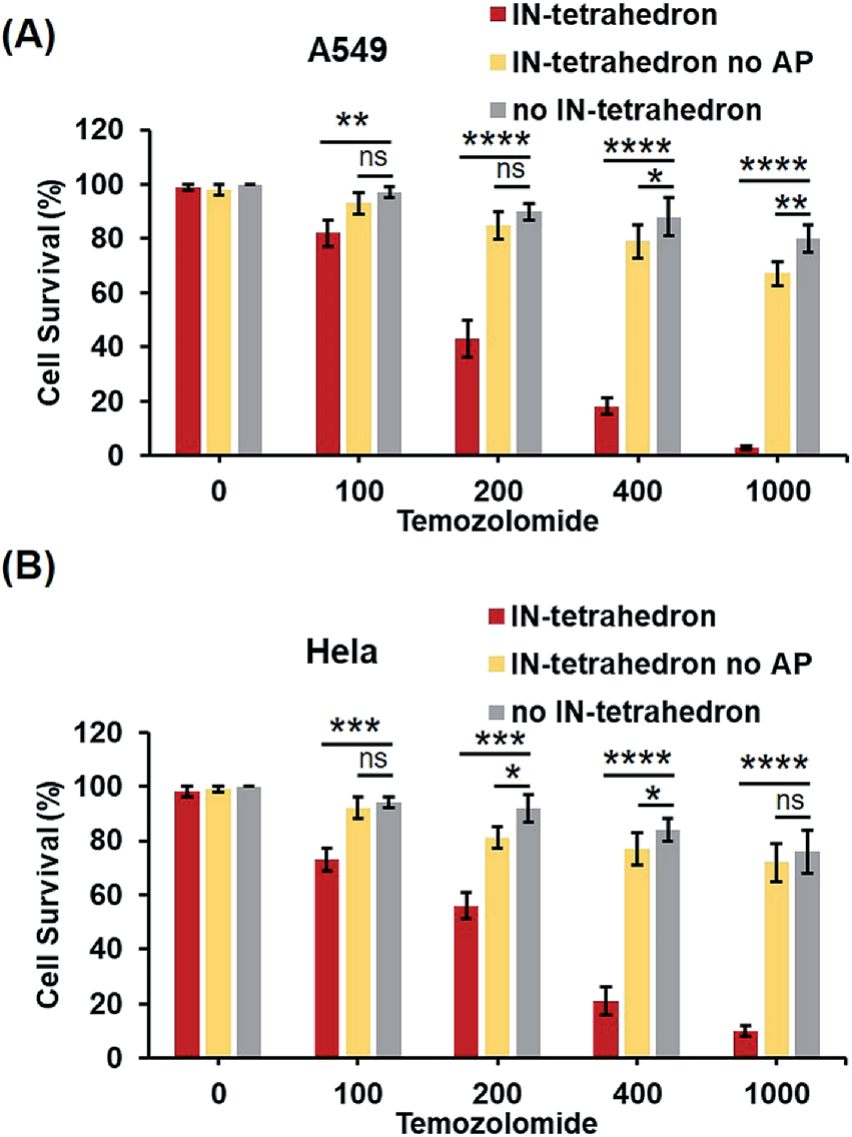
Potentiation of cytotoxicity of an anticancer drug (Temozolomide) by the IN-tetrahedron. The percentage survival of A549 cells (A) and HeLa cells (B). The gray columns represent the survival of the cells exposed to Temozolomide alone. The red and yellow columns represent the survival of the IN-tetrahedron (400 nM) and the IN-tetrahedron without the AP site (400 nM) pretreated cells exposed to Temozolomide, respectively. ns *p* > 0.05, *p < 0.05, **p < 0.01, ***p < 0.001, and ****p < 0.0001.

Previous biochemical and clinical studies confirmed that APE1 is an attractive target for anticancer drug development.^4^,^52^ Depletion of intracellular APE1 also sensitizes human cells to a variety of alkylating agents.^53^ The lethality of clinically used anticancer therapeutics particularly DNA damaging agents can be enhanced by a temporal decrease of activity in APE1. A variety of small-molecule APE1 inhibitors were investigated for potentiating anticancer drugs.^13^,^29^ However, small molecules are generally associated with multiple drug resistance and side effects. DNA tetrahedrons are an emerging tool for delivering various types of drugs such as small molecules and siRNA.^54^,^55^ Xie *et al.* utilized DNA tetrahedron structures successfully overcoming the drug-resistance of paclitaxe for lung cancer.^56^ But it is rarely reported that a DNA tetrahedron itself can be used as a drug or a drug enhancer. Given that the IN-tetrahedron exhibits a stronger inhibitory effect of APE1 than many small molecules, we speculated that it can sensitize cancer cells to drugs. To evaluate the potentiation of cytotoxicity of anticancer drugs by the putative APE1 inhibitor IN-tetrahedron, survival analyses were carried out in A549 and HeLa cells. Temozolomide as an alkylating agent was used for treatment of various types of cancers. As shown in Fig. 6A and B, both of A549 and HeLa cells are more sensitive to Temozolomide in the presence of the IN-tetrahedron than those in the absence of tetrahedrons or in the presence of the tetrahedrons without the AP site. This confirms that the drug potentiation is mainly attributed to the inhibition of APE1. The percentage survival of the IN-tetrahedron treated cells is lower than 10% under 1000 μM Temozolomide. It is noteworthy that the IN-tetrahedron does not exhibit cytotoxicity in the absence of Temozolomide. Fig. S9 ESI† shows the percentage survival of the two cell lines as a function of IN-tetrahedron concentration in the presence of Temozolomide. The dosage of the IN-tetrahedron is lower than that of previously reported small molecules.^11^–^13^

## Conclusions

In summary, we developed a novel approach for probing and regulating APE1 activity in living cells. Both the APE1 probe and inhibitor are DNA tetrahedron nanostructures. The location of the AP site on the tetrahedron determines the function of the nanostructure. The OUT-tetrahedron was used for probing the intracellular APE1 activity due to its high sensitivity and specificity to APE1. The IN-tetrahedron was utilized as an inhibitor of APE1 that regulates the APE1 activity in cancer cells because it exhibits high binding affinity and low reactivity to APE1. More importantly, the inhibitory effect of the IN-tetrahedron permits the potentiation of cytotoxicity of anticancer drugs. There is no doubt about the critical roles of APE1 in cancer biomarkers and druggable targets. We anticipate that this work would herald more applications in novel cancer therapeutics.

## Conflicts of interest

There are no conflicts to declare.

## Supplementary Material

Supplementary information
