# Supplementary material for: Probing and regulating the activity of cellular enzymes by using DNA tetrahedron nanostructures
Source: Chem Sci. 2019 May 6;10(23):5959–66. doi: 10.1039/c9sc01912j (PMC6566069; doi:10.1039/c9sc01912j)
Supplement: Supplementary file 1 [file SC-010-C9SC01912J-s001.pdf]

Electronic Supplementary Material (ESI) for Chemical Science.
This journal is © The Royal Society of Chemistry 2019

Electronic Supplementary Information

Probing and Regulating the Activity of Cellular Enzyme by DNA Tetrahedron Nanostructures

Yi Zhang,[a] Yingnan Deng,[a] Congshan Wang,[a] Lidan Li,[a] Lida Xu,[a] Yingjie Yu[b,*] and Xin Su[a,*]

[a] College of Life Science and Technology, Beijing University of Chemical Technology, Beijing 100029, China.

[b] Department of Biomedical Engineering, Tufts University, Medford, MA 02155, USA.

[*] Corresponding author

Email:

Y. Yu: yuyingjie312@outlook.com

X. Su: xinsu@mail.buct.edu.cn

Tel: +86-10-64421335

Fax: +86-10-64416248

Table of contents

Experimental Section

Sequence of the DNA used in this work in Table S1.

Supporting Figures 1-8.

Experimental Section

Reagents and materials. All of the oligonucleotides used in this work were synthesized and purified by HPLC (Sangon Co., Shanghai, China), their sequences are summarized in Table S1 and the use of these strands is explained in the denotation. APE1, lambda exonuclease (λ exo), deoxyribonuclease I (DNase I), exonuclease III (Exo III), exonuclease I (Exo I), T7 exonuclease (T7 Exo), T5 exonuclease (T5 Exo), and their corresponding buffers were obtained from NEB (Ipswich, MA). All chemicals were used as received without additional purification. DNase/RNase free deionized water from Tiangen Biotech Co. (Beijing, China) was used in all experiments.

Preparation and Characterization of the DNA Tetrahedrons. To prepare the DNA tetrahedrons, the oligonucleotides (final concentration of each strand 1 μM) were mixed in 1×TAE-Mg^{2+} buffer (40 mM Tris, 20 mM acetic acid, 12.5 mM $MgCl_2$, and 2 mM EDTA, pH 8). The above solution was denatured at 80°C for 10 min followed by cooling to 4°C in 30 min by using a PCR thermocycler. The nanostructures were purified by centrifugation at 12000 rpm for 10 min by ultrafiltration devices (Vivaspin, Sartorius, 30k, 0.5 mL). The nanostructures were re-suspended in the TAE-Mg^{2+} buffer. The non-labeled DNA nanostructures were characterized by 12.5% native PAGE which was operated at room temperature for 4 h at a constant voltage of 120 V. The gel was subsequently stained with SYBR Gold. To characterize the tetrahedron by AFM, a drop of 2 μL DNA solution was spotted onto freshly cleaved mica surface, and kept for 10 seconds to achieve strong adsorption. The sample drop was then washed off by 30 μL TAE-Mg^{2+} buffer, and dried by compressed air. DNA samples were imaged by tapping-mode AFM (FastScan, Bruker) with OTESPA-R3 tips. The tip-surface interaction was minimized by optimizing the scan set-point.

Characterization of the APE1 activity by fluorescence dequenching assay. All the in-vitro enzymatic reactions were carried out in 50 μL sealed PCR tubes. Once APE1 was added, fluorescence was recorded immediately in the FAM channel (ex: 480 nm, em: 510 nm) of a real-time PCR cycler (Rotor-Gene Q, QIAGEN, Germany) at 37 °C with a time interval of 5 s. 10 mM EDTA (final concentration) was used to terminate the reaction. The products were characterized by 12.5% native PAGE.

Cell culture and fluorescence imaging of the APE1 activity. A549, Hela and HEK-293 cell lines were cultured in 1640 medium supplemented with 1% Penn/Strep and 10% fetal bovine serum and incubated at 37 °C in a humidified atmosphere of 5% CO_2/95% air. The cells were

transferred to a laser confocal culture dish for fluorescence imaging in an appropriate density. The cells were incubated with 100 nM of DNA tetrahedrons (labeled with Cy3 and BHQ2) and other reagents in a low-fluorescence culture media (FluoroBrite DMEM, Thermo Fisher). Highly inclined and laminated optical sheet (HILO) fluorescence microscopy was constructed using a Nikon inverted microscopy (ECLIPSE, Ti−U) equipped with a 100×magnification, 1.49 numerical aperture (NA) TIRFM objective (Nikon) and an EMCCD cameras (ixon 897, Andor). For HILO illumination, the laser of 520 nm was coupled into a single-mode fiber (Solamere Technologies). The fiber optic cable that delivers laser light to the microscopy was secured into a fiber launch fitted with an XY fiber holder mounted atop a micrometer-driven optical rail for Z adjustment (Thorlabs). The pixel size of this camera matches very well with the magnification offered by the 100× TIRF objective, giving a final resolution of 0.15 μm per pixel. An optical filter (Semrock) of 590 nm was used to detect the emission of Cy3. The fluorescence images were acquired with an exposure time of 50 ms (gain 3).

Cell viability measurement by CCK-8 kit. The cells were seeded in a 96-well plate at a density of 10^4 cells/well in 100 μL of culture medium. To evaluate the potentiation of cytotoxicity of DNA damaging agents by IN-tetrahedron, various concentrations of IN-tetrahedron and Temozolomide were added to the medium and incubated with the cells for 24 h in the CO_2 incubator at 37 °C. 10 μL of CCK-8 solution was added and incubated for 2 h, then the O.D. at 450 nm was measured using a microplate reader (EnVision, PerkinElmer, UK).

Western blotting analysis of APE1. Cytoplasmic and nuclear fractions of the cells were obtained using NE-PER ExKine Nuclear and Cytoplasmic Protein Extraction Kit according to manufacturer's instruction manuals (Abbkine, KTP3001). The extracts were collected and stored at -80 °C for use. The proteins’ concentration were determined with BSA standard curve method from absorbance. For western blotting analysis, 20 μg of cytoplasmic proteins and 15 μg nuclear proteins were resolved on 10% SDS-PAGE, transferred onto nitrocellulose membranes, and probed with anti-APE1 antibodies (1:5000) (ab194, abcam). The membranes were incubated with goat anti-mouse IgG labeled with horseradish peroxidase (HRP) (1:5000) (ab6789, abcam). Bands on blots were visualized using enhanced chemiluminescence (ECL) detection system (5200, Tanon, China). α-tubulin and lamin b1 were used as reference for cytoplasmic protein and nuclear protein, respectively.

Table 1 Sequence of the oligonucleotides in this work

Name	Sequence (5'-3')
a1_AP	TGGGTAGTCA**X**CGCTACCACACACAGAACTCCCTACGACATTGGCATGAGATACTCGGAGACC
a1	TGGGTAGTCACCGCTACCACACACAGAACTCCCTACGACATTGGCATGAGATACTCGGAGACC
a1_AP_FAM_BHQ1	T(BHQ1)GGGTAGTCA**X**CGCT(FAM)ACCACACACAGAACTCCCTACGACATTGGCATGAGATACTCGGAGACC
a1_AP_Cy3_BHQ2	T(BHQ2)GGGTAGTCA**X**CGCT(Cy3)ACCACACACAGAACTCCCTACGACATTGGCATGAGATACTCGGAGACC
a2_AP	GCACTTCCAGCATGGGTAGTCA**X**CGCTACCACACACAGAACTCCCTACGACATTGGCATGAGATACTCGGAGACC
a2_AP_FAM_BHQ1	GCACTTCCAGCAT(BHQ1)GGGTAGTCA**X**CGCT(FAM)ACCACACACAGAACTCCCTACGACATTGGCATGAGATACTCGGAGACC
a2_AP_Cy3_BHQ1	GCACTTCCAGCAT(BHQ2)GGGTAGTCA**X**CGCT(Cy3)ACCACACACAGAACTCCCTACGACATTGGCATGAGATACTCGGAGACC
a2_Cy3_BHQ2	GCACTTCCAGCAT(BHQ2)GGGTAGTCAGCGCT(Cy3)ACCACACACAGAACTCCCTACGACATTGGCATGAGATACTCGGAGACC
b	GTCTCCGAGTATCTCATGCCCATTTGTGCCTAGGGTATCACGCTTTATCTGTAGCTCGCCCCC
c	GGAGTTCTGTGCTTGGATCACGCGTCGGTTGTTGTGATACCCTAGGCACAAATGATGTCGTAG
d1	GGGGCGAGCTACAGATAAAGAACAACCGACGCGTGATCCAATGTGGTAGCGCTGACTACCCAC
d2	GGGGCGAGCTACAGATAAAGAACAACCGACGCGTGATCCAATGTGGTAGCCTTGACTACCCAC
d3	GGGGCGAGCTACAGATAAAGAACAACCGACGCGTGATCCAATGTGGTAGTCTTGACTACCCAC
e	CGCGAGAGCGCTGACTACCCAGGCGTAGCCTCTACAGCTAGCGCGGCCACAGTGCGCTATCTGTTTCTACTACCGTCTACCACG

f	CACATGAAGACCTACTTCGCGGAGCATTGGACCGCTTTCTAGCGGCTAGCTGTAGAGGCTACG
g	CGTGGTAGACGGTAGTAGAACCGCTAAAGAGCCGCGGTACCCCGCGAAGTAGGTCTTCATGC
h	CACTGTGGCCGCGCTAGAAAGCGGTCCAATGCGGTACCCGCGGCTCTTTAGCGCCAGATAGCG
Input1	GTAGCGCTGACTACCCATGCTGGTTCACG
Input2	CGTGAACCAGCATGGGTAGTCAGCGCTAC
s1	ACT(FAM)AGTAG**X**ACTGCTCACAC(BHQ1)
s2	TGAGCAGTGCTACT

X represents the AP site.

Explanation the use of all strands.

DNA for *in vitro* assays:

IN-tetrahedron: a1_AP + b + c + d1

OUT-tetrahedron: a2_AP + e + f + g + h

FAM and BHQ1 labeled IN-tetrahedron with AP site: a1_AP_FAM_BHQ1 +b + c +d1

FAM and BHQ1 labeled OUT-tetrahedron with AP site: a2_AP_FAM_BHQ1 + e + f + g + h

FAM and BHQ1 labeled IN-tetrahedron with AP site and 1 mismatch: a1_AP_FAM_BHQ1 +b + c +d2

FAM and BHQ1 labeled IN-tetrahedron with AP site and 2 mismatches: a1_AP_FAM_BHQ1 +b + c +d3

dsDNA probe used in Figure 2: s1+s2

DNA tetrahedron and Inputs in Figure 3:

FAM and BHQ1 labeled IN-tetrahedron with AP site: a2_AP_FAM_BHQ1 +b + c +d1

Invader strands: Input1 + Input2

DNA for fluorescence imaging and cytotoxicity assay:

Cy3 and BHQ2 labeled IN-tetrahedron with AP site: a1_AP_Cy3_BHQ2 +b + c +d1

IN-tetrahedron: a1_AP + b + c + d1

Cy3 and BHQ2 labeled OUT-tetrahedron with AP site: a2_AP_Cy3_BHQ2 + e + f + g + h

Cy3 and BHQ2 labeled OUT-tetrahedron without AP site: a2_Cy3_BHQ2 + e + f + g + h

IN-tetrahedron without AP site: a1 + b + c + d1

Supplementary Figures

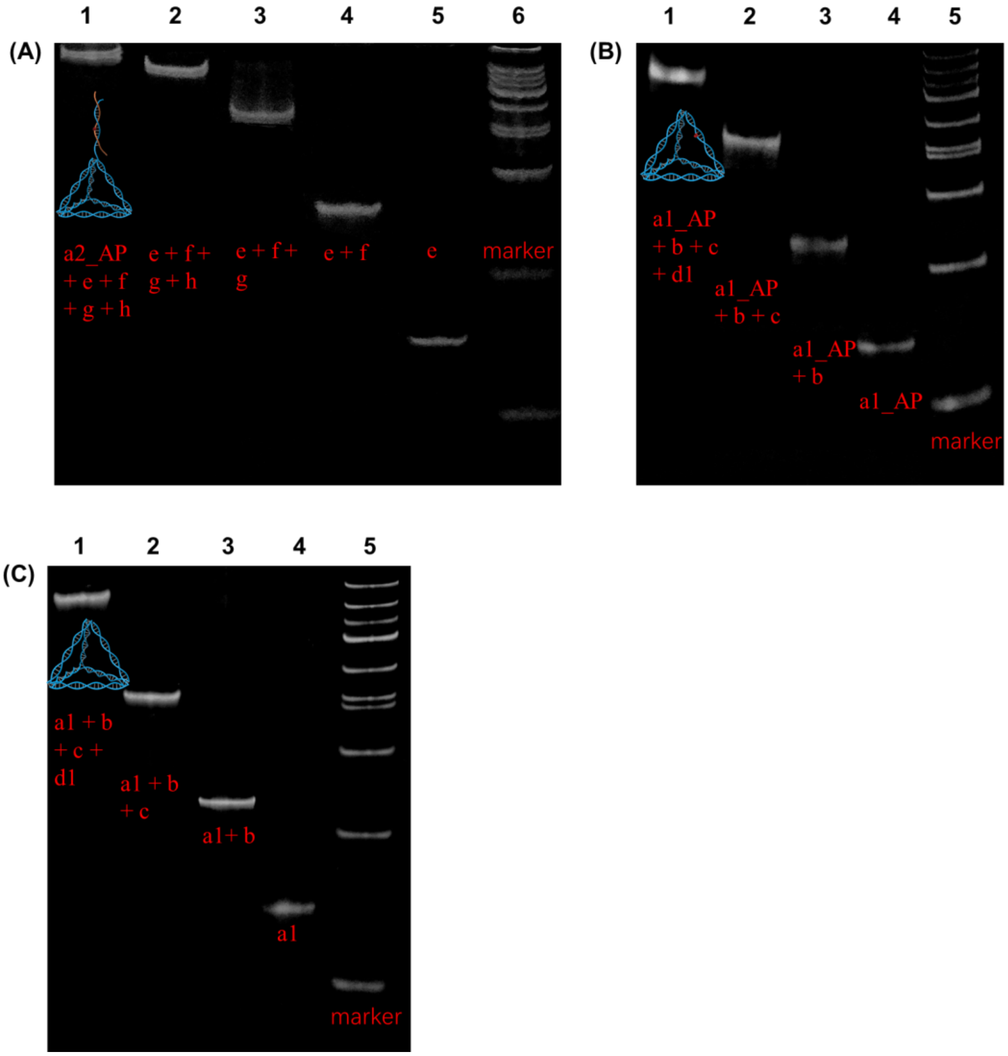

Fig. S1 Native PAGE gel (12.5%) for the DNA tetrahedrons, (A) OUT-tetrahedron, (B) IN-tetrahedron, (C) DNA tetrahedron without AP site (the same sequence as the IN-tetrahedron). The strands for each lane were marked (for sequence see Table S1).

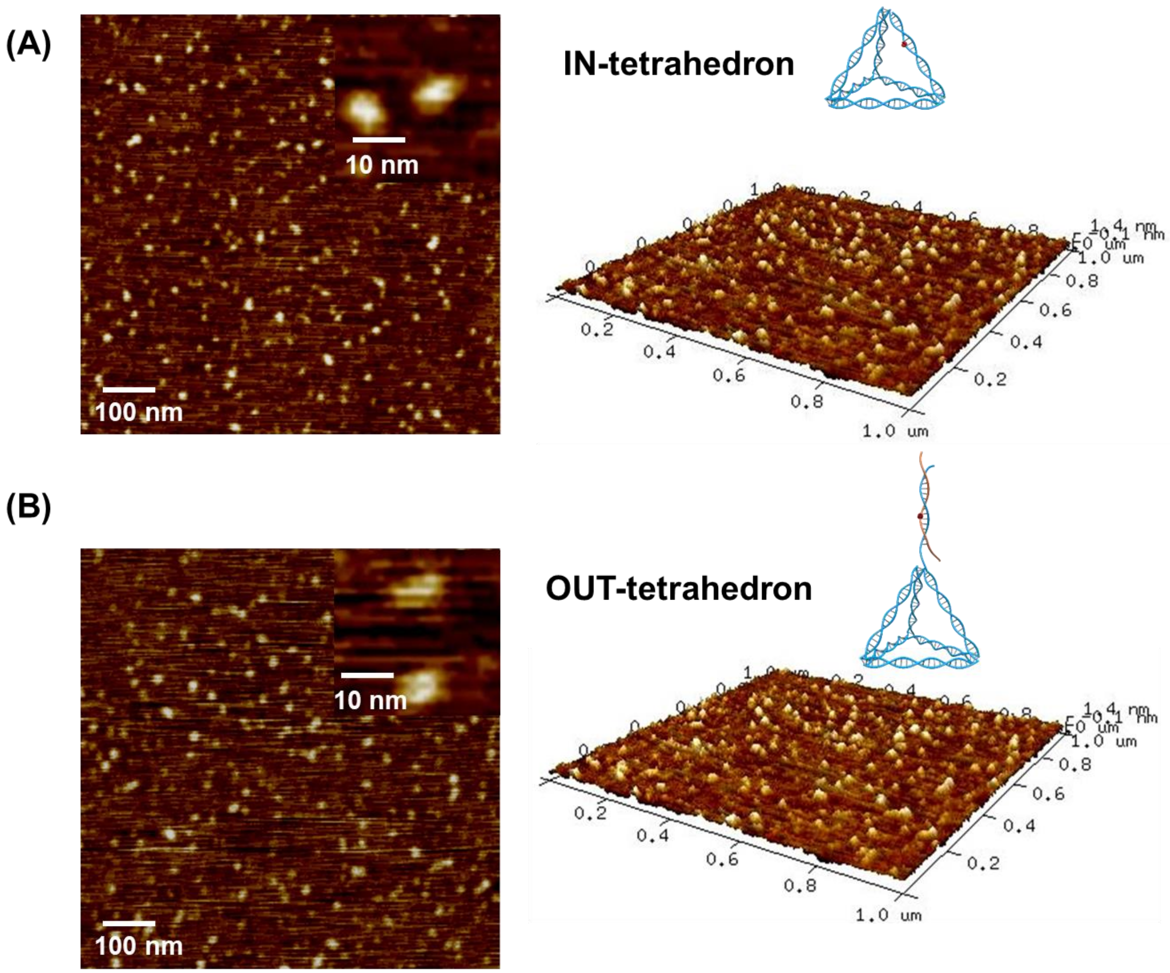

Fig. S2 AFM characterization of the DNA tetrahedrons, (A) IN-tetrahedron, (B) OUT-tetrahedron.

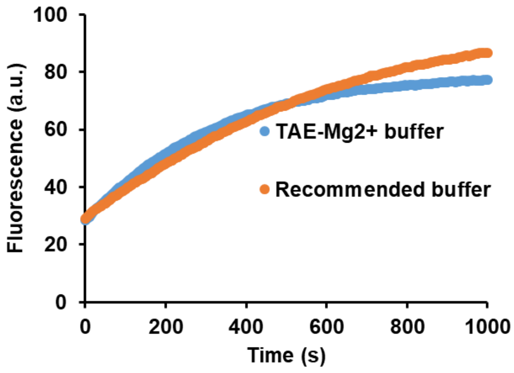

Fig. S3 APE1 activities in the TAE-Mg^{2+} buffer and the enzyme recommended buffer (50 mM Potassium Acetate, 20 mM tris-acetate, 10 mM magnesium acetate, 1 mM DTT, pH 7.9).

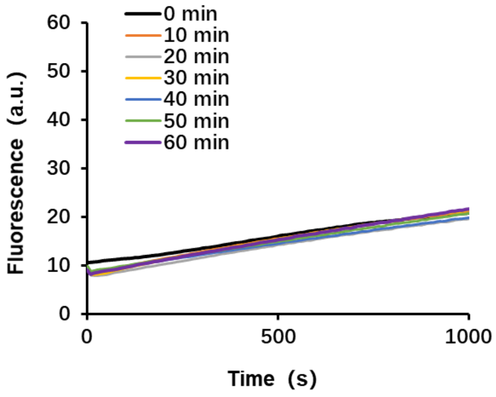

Fig. S4 Raw fluorescence signals of the dsDNA probe digested by IN-tetrahedron bound APE1 (pre-assembled for different time).

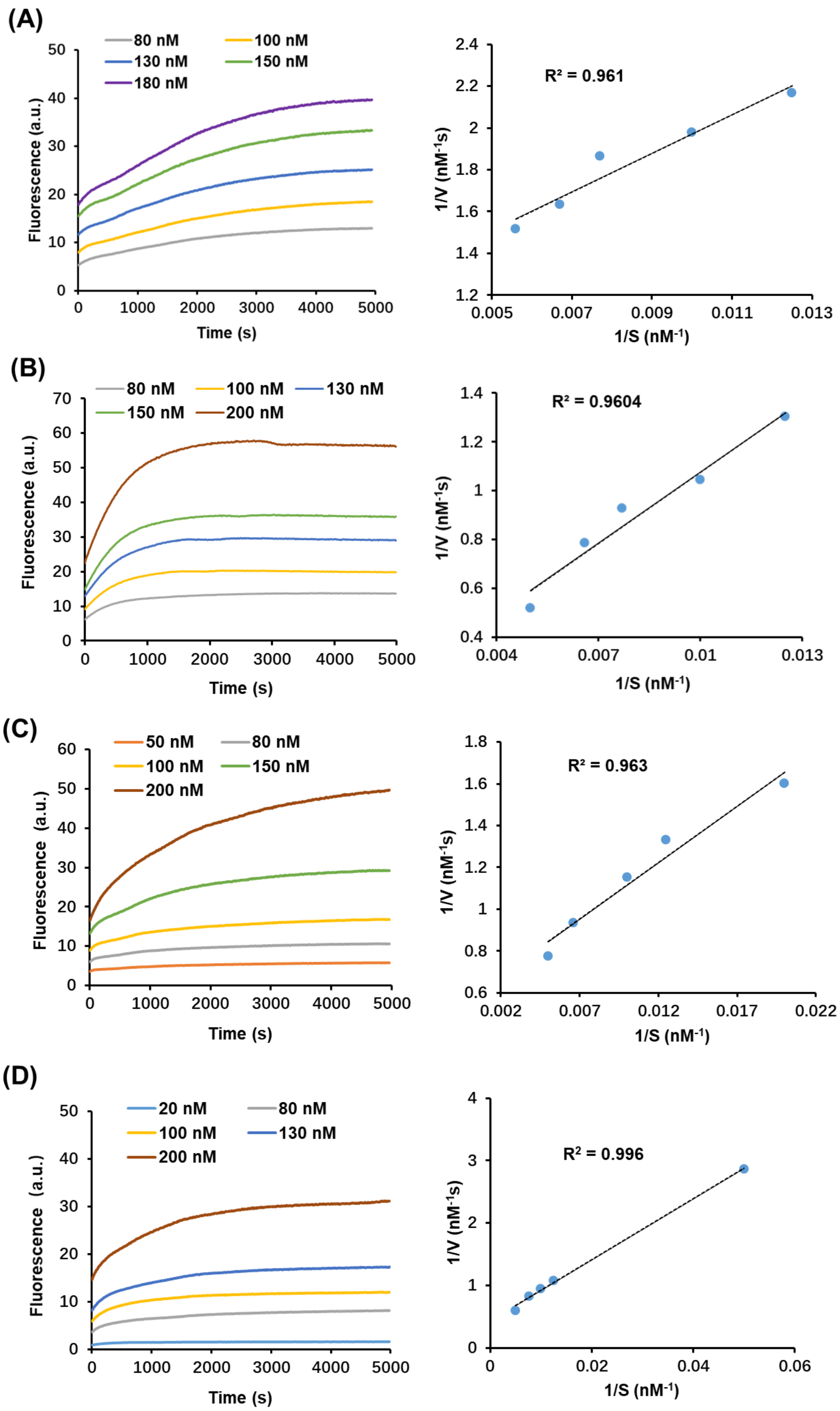

Fig. S5 The raw fluorescence curves (left panel) and Lineweaver-Burk plots (right panel) of the APE1 kinetics towards different substrates, (A) IN-tetrahedron, (B) OUT-tetrahendron, (C) IN-tetrahedron 1 mismatch, (D) IN-tetrahedron 2 mismatches.

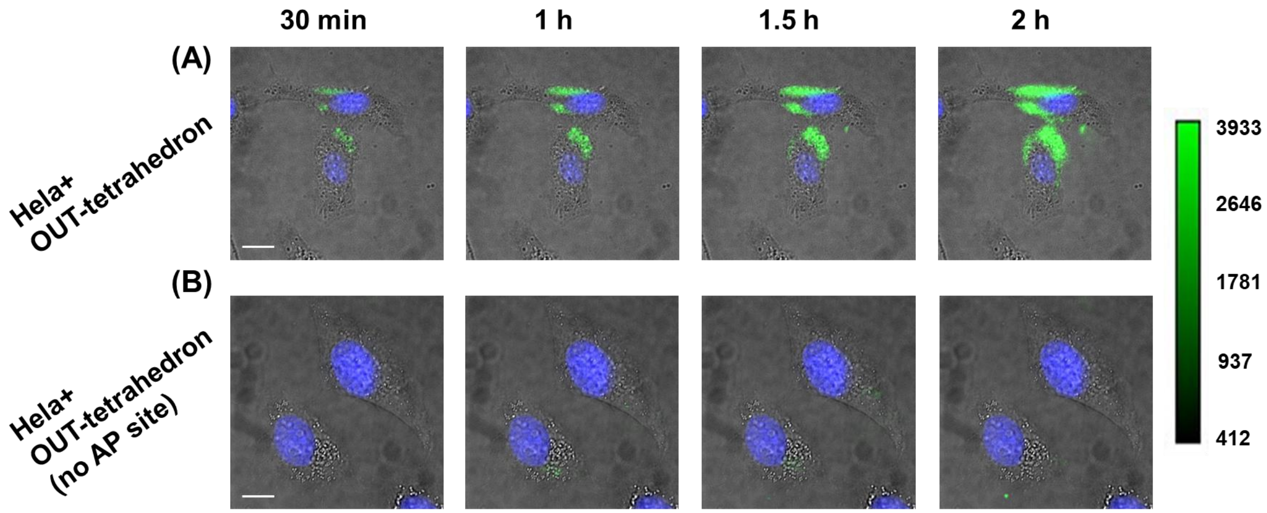

Fig. S6 Fluorescence imaging of the Hela cell with OUT-tetrahedron at different time, (A) AP site, (B) No AP site. Scale bar: 5 μm. The nucleus was stained by Hoescht33342.

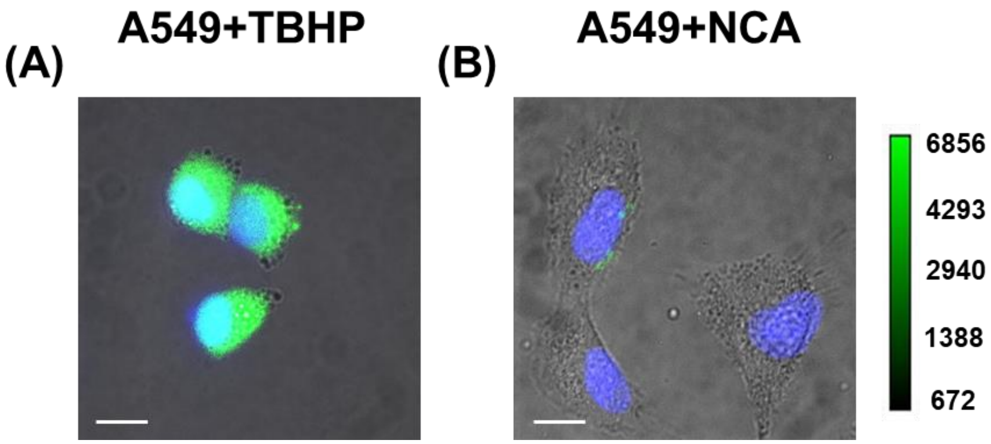

Fig. S7 Fluorescence imaging of the TBHP (A) and NCA (B) treated A549 cell with OUT-tetrahedron. Scale bar: 5 μm. The nucleus was stained by Hoescht33342.

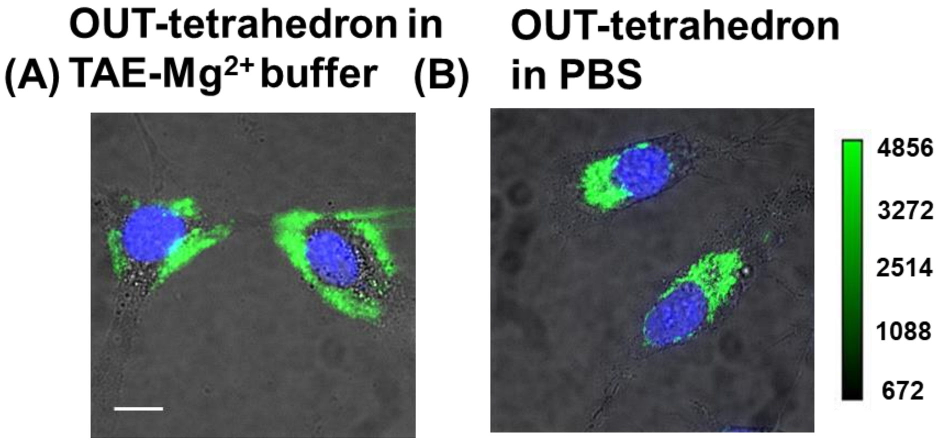

Fig. S8 Comparing the tetrahedrons in the TAE-Mg^{2+} buffer (A) and in 1×PBS (B) by the assay of Fig. 5A. Scale bar: 5 µm. The nucleus was stained by Hoescht33342.

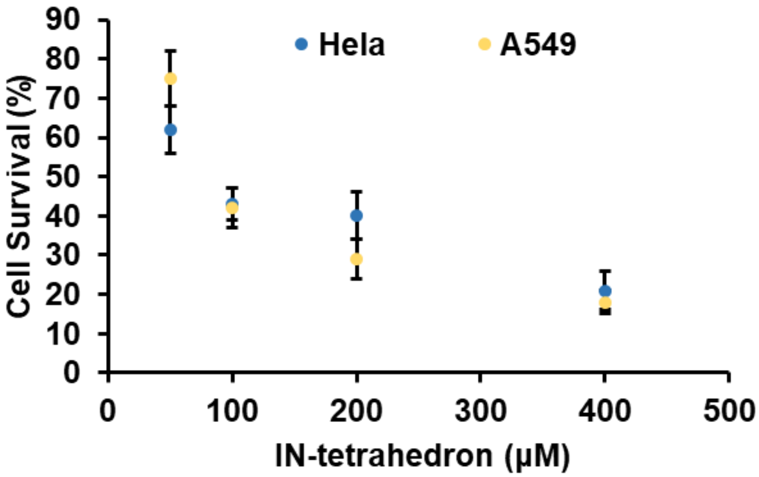

Fig. S9 The percentage survival of A549 cell (yellow) and Hela cell (blue) as a function of IN-tetrahedron concentration in the presence of 400 nM Temozolomide.
